# The benefit of an ambulant psychiatric rehabilitation program in Vienna, Austria: an uncontrolled repeated measures study

**DOI:** 10.1007/s10100-021-00773-2

**Published:** 2021-10-09

**Authors:** Alexandra Schosser, Birgit Senft, Marion Rauner

**Affiliations:** 1 Department of Psychiatry and Psychotherapy, Medical University of Vienna, Währinger Gürtel 18-20, 1090 Vienna, Austria; 2 Zentren für seelische Gesundheit, BBRZ-Med, Schererstrasse 30, 1210 Vienna, Austria; 3 Faculty of Medicine, Sigmund Freud University, Freudplatz 3, 1020 Vienna, Austria; 4 Arbeitsgemeinschaft für Verhaltensmodifikation, Paris-Lodron-Straße 32, 5020 Salzburg, Austria Birgit Senf Vienna, Austria; 5 Faculty of Business, Economics, and Statistics, Institute for Business Decisions and Analytics, University of Vienna, Oskar-Morgenstern-Platz 1, 1090 Vienna, Austria

**Keywords:** Ambulant psychiatric rehabilitation, Vienna, Austria, Uncontrolled repeated measures study, Statistical analysis, Effectiveness analysis, Cost analysis

## Abstract

We investigated the benefit of a 6-week ambulant psychiatric rehabilitation program in an ambulant psychiatric rehabilitation clinic in Vienna, Austria, from January 2014 to December 2016 by an uncontrolled repeated measures study. The potential of this intervention program was assessed by effectiveness and cost measures using suitable statistical analyses. We compared the effectiveness and cost measures of this ambulant psychiatric rehabilitation program on patients for the period of up to 12 months after discharge to the period of 12 months before admission to the intervention program based on self-reported catamnesis questionnaires. For the program’s effectiveness measures, we accounted for both psychological indices for measuring depression severity, symptom burden, and functioning to document the health improvement of patients and economy-related indices such as the number of sick leave days for patients. For the program’s cost measures, both direct tangible treatment and medication costs and indirect tangible costs based on the productivity loss measured in non-working days of the patients were considered. The results significantly demonstrated that all psychological effectiveness measures for the patients highly improved by the 6-weeks rehabilitation program and remained rather stable 12 months after discharge. We found that costs for the 6-week ambulant psychiatric rehabilitation program could be easily covered within 12 months after discharge once a total societal cost perspective was considered. Even additional total cost savings of up to over 5000 Euro could be achieved which were highest for employed patients, followed by unemployed patients receiving rehabilitation allowance due to both their high direct medication and treatment costs as well as high indirect costs for productivity loss. The most important finding was that this treatment program was especially beneficial for rehabilitation patients in earlier stages of psychiatric diseases who were still employed, indicating the need for early intervention in mental disorder.

## Introduction

In the current times, humans and especially the productive workforce of countries are exposed to an unprecedented pressure resulting in overwork, burn-outs, as well as chronical and psychological diseases (World Health Organization 2013; Atroszko et al. 2020; Sunkel 2021). The underlying reasons for this trend are manifold such as demographical (e.g., single households, single parents, family structures), economical (e.g., low income, unemployment, state of economy), technological (e.g., rationalization, digitalization), and individual (e.g., life style, perspective of life, overreaching self-ambitions). This is why psychological diseases have sharply increased in the last years and also cause a high economic burden to countries worldwide (Knapp and Wong 2020; Sunkel 2021). Before the COVID (Corona Virus Disease)-19-pandemic had hit the world (Kola 2020), more than 450 million people have had some type of mental disorders (Saanich News 2018). About 90% of the suicides have been related to mental disorders. During lifetime, about one in four people has suffered from mental disorders. Direct and indirect costs of mental illness have amounted to over 2.5 trillion US-$ worldwide. However, health care policy makers have only spent about 3% of the health care budget for mental health needs. Although, the impact of mental disorders is high, the health care prioritization has been unfortunately still low worldwide (World Health Organization 2013; Konnopka and König 2020; Sunkel 2021).

The quality of life for untreated depressed people is generally worsening during the course of the disease. In most cases, people with mental disorders have a chronical course of illness and suffer from long duration of unemployability at the beginning of the therapy due to unspecific diagnoses (Rössler 2006; Barrett et al. 2013; World Health Organization 2013). Such mentally ill patients are longer on sick leave without obtaining a sound therapeutic concept for treating especially their depression. This is why, their mental disease will become chronic. These patients decrease their activity level and might lose their day structure. Furthermore, they might become more depressed and desperately need help by focused psychological treatment (Reiter et al. 2012; Bundesministerium für Arbeit, Soziales, Gesundheit und Konsumentenschutz 2019). After early mobilization in the intramural or extramural sector (phase 1), these mentally ill patients urgently need psychological rehabilitation in special facilities for several weeks (phase 2, either ambulant or inpatient treatment) before ambulatory care will then stabilize them in phase 3 and these patients might finally be cured in phase 4 (World Health Organization 2013).

Similar as in other countries, also in Austria, mental disorders are a high social and economic burden for the society (Bundesministerium für Arbeit, Soziales, Gesundheit und Konsumentenschutz 2019). For example, in 2018, psychiatric diseases were accountable for the majority of about 35% of the occupational disability cases leading to new premature pension cases due to invalidity. Austrian females were more affected compared to males. Diseases of the musculoskeletal system only caused about 21% of the new occupational disability cases, while cardiovascular diseases affected about 11% of the new cases. Therefore, there is a high need for efficient and effective psychological rehabilitation programs in special psychiatric health care facilities to support these mental patients to quickly recover from their illness and to be quickly reintegrated in the working process (Rössler 2006; Barrett et al. 2013; World Health Organization 2013).

This is why, among others reasons, health care policy makers established the first ambulant psychiatric rehabilitation clinic in Vienna, Austria, in 2010. This statistical study investigates both clinical effectiveness and cost measures associated with a 6-week ambulant psychiatric rehabilitation program at the “Zentrum für seelische Gesundheit BBRZ (Berufliches Bildungs- und Rehabilitationszentrum)-Med Wien-Leopoldau” for all patients from January 2014 to December 2016 by an uncontrolled repeated measures study (Sullivan 2008). We compared the success of this psychiatric rehabilitation intervention program for all patients during the period of up to 12 months after discharge compared to the period of 12 months before admission based on self-reported catamnesis questionnaires (Senft et al. 2020).

In the literature, most studies on psychiatric rehabilitation focused on clinical effectiveness measures. For example, the most comprehensive study of the effectiveness of inpatient rehabilitation programs in German-speaking countries was the MESTA (Meta-analysis of the effects of inpatients psychosomatic rehabilitation) study by Steffanowski et al. (2007), which found significant improvements of symptomatology measured by the 90-R Symptom Checklist (SCL-90-R) to assess psychological symptoms and psychopathological features of mentally ill patients (Franke 2000). Furthermore, a review on mental rehabilitation programs in prisons could only find modest improvement of patients due to their disease severity and late onset of the therapy (Yoon et al. 2017). Another literature review on community-based interventions for schizophrenia and other psychotic disorders reported both positive psychological effects after the first psychotic episodes and in other phases of the illness (Armijo et al. 2013). Similar findings were gathered by Na et al. (2016) for mentally ill patients with early psychosis receiving community mental health care services.

Moreover, several mental intervention programs for patients also reported improvements in economically relevant outcome measures as well, including a significant reduction in the number of sick-leaves, doctor visits, and hospital stays. In Austria, a study published by Haberfellner et al. (2008) showed significant reductions of mental disease symptomatology applying the Brief Symptom Inventory 18 items (BSI-18) score to assess the psychological distress and comorbidities in patients with different mental and somatic diseases, as well as reductions in both the number of sick leaves and hospital stays when performing a follow-up survey one year after the end of treatment. In addition, Rabenstein et al. (2015) found a significant reduction of symptomatology in mentally ill patients after 6 weeks ambulant rehabilitation treatment as assessed by the clinical scores BSI-18, BDI (Beck Depression Inventory) (Hautzinger 1991), and WHOQOL-Bref (World Health Organization Quality of Life, short version) (Saxena 2001). A recent review by Knapp and Wong (2020) discussed further economic intervention studies related to mental health worldwide. They reported that they found about 100 comprehensive studies in 1999, while over 4000 such studies were reported in 2019. Especially, maternal mental health care is a most valuable field with enormous impact both on mothers and their offspring as illustrated by a review of Camacho and Shields (2018). Furthermore, Knapp and Wong (2020) reported that the field of child and adolescent mental health also needs more attention to economic evidence in the medical studies untertaken (cf. e.g., Beecham 2014; Patton 2016). König et al. (2020) summarized in their systematic review that major depressive disorders have played a key role in cost-of-illness studies and have caused high health care expenditures worldwide.

Therefore, there is a high need for comprehensive mental health-related economic studies for policy makers to curb the corresponding exploding costs in all countries over the world. Hereby, quantitative operations research studies have played a critical role to manage scarce and limited ressoures as illustrated for advanced health care management studies in other fields (cf. e.g., Rauner and Vissers 2003; Brandeau et al. 2004; Rauner et al. 2005; Brailsford and Harper 2008; Brailsford and Vissers 2011; Zaric 2013; Weber et al. 2014; Morton et al. 2016; Malor et al. 2018).

For Austria, this study is the first statistical study which investigated both the clinical and economical effectiveness measures as well as cost measures for an ambulant psychiatric intervention program directed to mentally ill patients. We aim at illustrating to Austrian health care policy makers how effective such ambulant psychiatric intervention programs will be, if they are early intitiated especially to psychiatric rehabilitation patients who are still employed, that is before falling out of the job market. Health care policy makers should be motivated to invest more budget in mental health care in the future due to the increasing needs of the population, especially in times of COVID-19 (Kola 2020).

In Sect. 2, we explain the underlying methods of the study such as study design, outcome analysis regarding effectiveness and cost measures, and statistical analysis. The results of our study are presented in Sect. 3, whereas we first evaluate the impact of the mental ambulant psychiatric rehabilitation program on health-related effectiveness measures and then focus on cost-related measures. Section 4 concludes all important findings of this study and demonstrates selected key policy implications for ambulant psychiatric rehabilitation in Austria.

## Method

### Study design

**Fig. 1:**
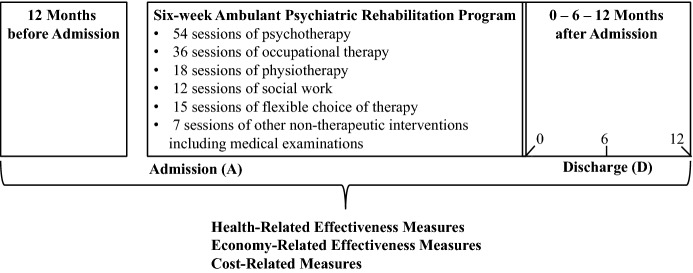
Design of the uncontrolled repeated measures study for the 6-week ambulant psychiatric rehabilitation program which was investigated in Vienna, Austria from 2014 to 2016 (adapted and updated based on Schosser 2017)

We investigated health care-related and economy-related effectiveness measures as well as cost-related measures of a specific 6-week multimodal ambulant psychiatric rehabilitation program in Vienna, Austria at the Zentrum für seelische Gesundheit Wien-Leopoldau from January 2014 to December 2016 as illustrated in Fig. 1 (adapted and updated based on Schosser 2017). The initial sample included in total 2486 patients. Repeated measurements were conducted by questionnaires for each rehabilitation patient without incorporation of a control group. For this study, we used one pre-therapy time point at admission (*A*) and up to three post-therapy time points after discharge (*D0*: at discharge, *D6*: 6 months after discharge, and *D12*: 12 months after discharge) depending on the outcome measures and the return rate of the questionnaires. At the time point of admission (*A*), we questioned the patients on their medical treatment 12 months before admission to the ambulant psychiatric rehabilitation program. Patients with missing data and patients, who discontinued the rehabilitation program, were excluded from further analyses, resulting in a final sample size of up to 1781 patients depending on the specific outcome measure. Our study belongs to the category of uncontrolled repeated measures studies (Sullivan 2008).

### Intervention program

In Austria, medical rehabilitation has been a duty of the pension insurance (32nd Allgemeines Sozialversicherungsgesetz Novelle) for such cases where impairment would lead to incapacity to work without rehabilitation since 1992. Thus, psychiatric rehabilitation is mainly funded by the pension insurance. Psychiatric rehabilitation has been available in Austria since 2002, initially only as inpatient treatment, and since 2010 as ambulant rehabilitation as well (Schosser 2017).

This 6-week multimodal ambulant psychiatric rehabilitation program was a phase 2 psychiatric rehabilitation program at a special ambulatory treatment center (Reiter et al. 2012) as explained in the introduction. Our phase 2 6-week ambulant psychiatric rehabilitation program consisted of 142 treatment sessions of 50 min each (cf. Fig. 1) within a period of 6 weeks including: (1) 54 sessions of psychotherapy, (2) 36 sessions of occupational therapy, (3) 18 sessions of physiotherapy, (4) 12 sessions of social work, (5) 15 sessions of flexible choice of therapy, and (6) 7 sessions of other non-therapeutic interventions including medical examinations.

### Outcome analysis regarding effectiveness and costs

To assess the effectiveness of this 6-week ambulant psychiatric rehabilitation program, we investigated both health-related (cf. Sect. 2.3.1) and economy-related effectiveness measures (cf. Sect. 2.3.2). Furthermore, we also calculated main cost-related measures (cf. Sect. 2.3.3) from a societal perspective as illustrated in Fig. 1.

#### Health-related effectiveness measures

Firstly, we considered health-related psychological indices as suitable effectiveness measures to document the potential of health care improvement after discharge compared to admission for up to 1781 rehabilitation patients (cf. Sect. 3.2).

Clinical effectiveness was measured by symptom reduction and/or increased functioning of rehabilitation patients. The scales used for clinical effectiveness measurement included:

1. the Beck Depression Inventory (BDI) (Hautzinger 1991),
2. the Brief Symptom Inventory-18 items (BSI-18) (Franke 2000; Derogatis and Melisaratos 1983),
3. the World Health Organization Disability Assessment Schedule 2.0 (WHODAS 2.0) (Üstün et al. 2010), and
4. the International Classification of Functioning, Disability and Health, Activities and Participation, 3 Factors (ICF-AT-3F) (Nosper 2008; Senft et al. 2013).

The scale and scope of these indices are discussed in detail in the results Sect. 3.2. In addition, we accounted for other economy-related improvement measures such as the number of days of medical treatment of the mentally ill patients before and after the ambulant psychiatric rehabilitation program. We did not account for quality-adjusted life years (QALYs) as an additional effectiveness outcome measure because long-term effects of this intervention strategy were not considered beyond 12 months after discharge of the patients and the focus of this study was on patients 12 months after discharge for whom all essential data were available. Therefore, we did not investigate the effect of this intervention program on life years or QALYs which could be further research.

#### Economy-related effectiveness measures

Secondly, we also investigated economy-related outcome measures of the ambulant psychiatric rehabilitation program for a sub-sample of 389 patients who had returned the questionnaires 12 months after discharge (cf. Section 3.2.5) such as: (1) employment status, (2) number of sick leave days, and (3) number of medical treatment days. These outcome measures after discharge of the mental patients can be compared to the related ones before admission.

Regarding the employment status, mentally ill patients were divided into the following three occupational groups: (1) employed (full-time or part-time), (2) unemployed, without receiving rehabilitation allowance, and (3) unemployed, receiving rehabilitation allowance (Sozialrechts-Änderungsgesetz 2012). A patient receives unemployment benefit during the period of working capacity, while a patient may obtain a so called “rehabilitation allowance (i.e., Rehageld)” in case of not being able to work due to health issues for more than 6 months. The more unemployed patients returned to employment after the mental intervention program and/or the more patients became capable for working, the higher was the success of the mental intervention program.

The outcome measure “number of sick leave days” is related to the level of productivity of a mentally ill patient during the period of 12 months after discharge. It can be compared to the similar measure for the period 12 months before admission to the intervention program. The lower the number sick leave days reported after discharge of patients was, the more successful the mental intervention program was.

Furthermore, patients reported on their medical treatment data during the period of 12 months after discharge (number of doctor visits, psychotherapy and/or psychology sessions, inpatient treatment, and pharmacological treatment) which we again compared with their reported medical treatment data during 12 months before admission to the intervention program. The medical treatment effort after discharge of patients was, the higher the effectiveness of the mental intervention program was.

#### Cost-related Measures

We calculated *main* *total costs* **(*C***_***total***_**)** from the economy-related effectiveness measures from a societal perspective *for the period of 12 months before admission* **(*C***_***total,adm***_**)** and *for the period of 12 months after discharge to the 6-week ambulant psychiatric rehabilitation program* **(*C***_***total,dis***_**)**. For this cost comparison, both *main tangible direct costs* (***C***_***direct,adm/dis***_**)** and *main tangible indirect costs* (***C***_***indirect,adm/dis***_) were considered (cf. Sect. 3.3). Transfer payments such as pension payments and sickness benefit were not included similar to the criteria for a cost-effectiveness analysis (Schöffski and von der Schulenburg 2008). Transfer payments do not belong to the indirect costs incurred by payments for resource needs because they serve as socio-political reallocation. In further research, they could be accounted for as an own category for transfer payments once these data could be provided by the social security system. As some cost data were not directly available per patient, average costs had to be calculated because such data were not publicly available for research in Austria. In addition, average *total costs per patient for the 6-week ambulant psychiatric rehabilitation program* (***C***_***rehab***_) were calculated by the average number of sessions consumed multiplied by the average costs per session which amounted to 3731.74 Euro to 4390.23 per patient.

A positive economic effect of the 6-week ambulant psychiatric rehabilitation program is also reflected by a reduction in the total costs for the period of 12 months after discharge **(*C***_***total,dis***_ **=** ***C***_***direct,dis***_ **+** ***C***_***indirect,dis***_**)** compared to the period of 12 months before admission to the intervention **(*C***_***total,adm***_ **=** ***C***_***direct,adm***_ **+** ***C***_***indirect,adm***_**).** If this difference in total costs (***C***_***total,adm***_ – ***C***_***total,dis***_) is equal or higher compared to the total costs for the 6-week ambulant psychiatric rehabilitation for a patient **(*C***_***rehab***_**)**, then direct treatment costs for the 6-week ambulant psychiatric rehabilitation of a patient could be even covered within 12 months after discharge.

We incorporated the following *main tangible direct costs* for the period of 12 months before admission (***C***_***direct,adm***_) and after discharge (***C***_***direct,dis***_**)** regarding the 6-week ambulant psychiatric rehabilitation program for each patient (all cost components are displayed in Table 1):

- average visit costs for ambulant treatment by general practitioner/psychiatrist/psychotherapist/psychologist (per visit multiplied by the number of visits),
- average costs for inpatient treatment (per diem multiplied by the days of treatment), and
- average costs for psycho-pharmacological treatment (annual).

**Table 1:** Main tangible direct cost components (***C***_***direct,adm/dis***_**)** for psychiatric patients by category for the 12 month period before and after admission (adapted based on Schosser 2017)

**Direct Cost Components** (*C*_*direct,adm/dis*_)	Average Costs in Euro (Baseline)	Source
**Per visit costs for ambulant treatment by specialists**		
General practitioner	75 Euro	Schneider and Dreer (2013)
		Wiener Gebietskrankenkasse (2016)
Psychiatrist	150 Euro	Schneider and Dreer (2013)
		Wiener Gebietskrankenkasse (2016)
Psychotherapist	110 Euro	bestNET Information-Service GmbH (2016)
Psychologist	170 Euro	Sigmund Freud University (2016)
**Per diem costs for inpatient treatment**	736.20 Euro	Landeskrankenhaus Steyr (2016)
**Annual costs for psycho-pharmacological treatment**	150 Euro	Schneider and Dreer (2013)

In addition, we included the *main tangible indirect costs* (***C***_***indirect,adm/dis***_) which we derived from the economy-related effectiveness measures. We calculated the productivity loss for each patient in Euro measured in non-working days multiplied by the average daily income of each patient depending on work sector, gender, age, and scope of employment (full-time work, part-time work). For the categorization of the work sector, we used the ÖNACE (Austria’s national version [Ö, Österreich] of the NACE, Nomenclature statistique des activités économiques dans la Communauté européenee) 2008 classification system (Statistik Austria 2019). We obtained these income data by the Statistik Austria (2017).

### Statistical Analyses

Statistical analyses were performed using IMB SPSS Statistics Version 23 (https://www.ibm.com/products/spss-statistcs) (IBM Corporation 2017). To identify significant differences concerning means of metric parameters of different patient groups, paired samples T-tests (two time-points) and ANOVA (Analysis of Variance, more than two time-points) tests were computed in case of normality, whereas non-parametric related-samples Wilcoxon Signed Rank tests (Wilcoxon 1945) were performed if requirements for parametric test statistics were not met.

ANOVA analyses were performed to identify different distributions (means) among the patient sub-groups or categories of an independent variable. The effect sizes were calculated according to Cohen (1988, 1992). Cohen’s d below 0.2 was assumed as a small effect, around 0.5 as a medium effect, and around or above 0.8 as strong effect (Lenhard and Lenhard 2016). For the ANOVA analyses, effect sizes were calculated as partial eta squared (*η2*), corresponding an effect size of *f* (small effect size: *f* = 0.10, medium effect size: *f* = 0.25, large effect size: *f* = 0.40). Please note that the Cohen's effect size indicates the ratio between the difference of means in the two groups which are compared and the related standard deviation.

## Results

After explaining the subject characteristics of the study in Sect. 3.1, we present our evaluation of the health-related, clinical effectiveness measures (cf. Sect. 3.2) and then of the cost-related measures (cf. Sect. 3.3) regarding the mentally ill patients before and after the 6-week ambulant psychiatric rehabilitation program.

### Subject characteristics

The characteristics of the 1781 patients in the ambulant psychiatric rehabilitation program of the time of discharge from 2014 to 2016 in Vienna, Austria are shown in Table 2. This uncontrolled repeated measures study was conducted at the “Zentrum für seelische Gesundheit BBRZ-Med, Wien-LEOpoldau” (adapted and updated based on Schosser 2017). The number of patients treated per annum was rather equally distributed over the three years investigated. The patients were predominantly female (65.7%), since two thirds of patients suffer from a depressive disorder (ICD-10: F3) that occurs twice as often in females than in males (Cyranowski et al. 2000), with a mean age of 43.8 years (SD 9.65).

Primary diagnoses according to International Classification of Disease 10th revision (ICD-10) (World Health Organization 1993) at time of discharge were mainly affective (66.7%) [group F3] or anxiety disorders (22.7%) [group F4], while other diagnoses included schizophrenia spectrum disorders (4.2%) [group F2] or personality disorders (5%) [group F6]. A total of 38.4% of the patients had one psychiatric diagnosis, while 41.7% obtained two psychiatric diagnoses. About 15.2% had even three and 4.7% had even four diagnoses, respectively.

Data concerning the occupational status were available for 1696 patients at admission/discharge. A total of 28.7% of the patients were employed, whereas 73.9% worked full-time and 20.1% part-time (no information available for the remaining 6%). In addition, 63.4% were unemployed, without receiving rehabilitation allowance and 7.8% were unemployed, receiving rehabilitation allowance. Most patients were unemployed due to a high number of absent days from work and/or incapability of work because of their disease. The aim of the 6-week psychiatric rehabilitation program is to maintain capacity to work, reintegrate unemployed patients to the job market and to improve the health status of the patients which can be measured by health-related, clinical effectiveness measures after discharge (cf. Sect. 3.2).

**Table 2:** Characteristics of the 1781 patients in the 6-week ambulant psychiatric rehabilitation program at the time of discharge from 2014 to 2016 in Vienna, Austria (adapted and updated based on Schosser 2017)

**Characteristics of the Study Sample**	Number	%
*Total Number of Patients*	1781	100.00
*Admission Year*		
2014	639	35.9
2015	588	33.0
2016	554	31.1
*Gender*		
Female	1171	65.7
Male	610	34.4
*Age*		
15–19 years	5	0.3
20–24 years	180	10.1
25–34 years	365	20.5
35–44 years	625	35.1
45–54 years	593	33.3
55–59 years	13	0.7
Occupational status^*^		
Employed	487	28.7
Unemployed (without receiving rehabilitation allowance)	1076	63.4
Unemployed (receiving rehabilitation allowance)	133	7.8
*Main Four Primary Psychiatric Diagnoses (Based on ICD-10)*		
F3: mood disorders	1188	66.7
F4: neurotic, stress-related and somatoform disorders	405	22.7
F6: disorders of adult personality and behavior	89	5.0
F2: schizophrenia, schizotypal and delusional disorders	75	4.2
*Number of Psychiatric Diagnoses*		
One	684	38.4
Two	742	41.7
Three	271	15.2
Four	84	4.7

*The employment status was only available for 1696 patients

### Evaluating the health-related, clinical effectiveness measures

The health-related, clinical effectiveness of the 6-week ambulatory psychiatric rehabilitation program was measured by symptom reduction and/or increased functioning of the patients at discharge (*D0*) and 6/12 months after discharge (*D6*/*D12*). The better the health condition of a patient is, the higher is the chance of being employed and the lower the days of treatment as well as the number of days of sick leave. This is both beneficial for patients and the society.

The clinical scales used for clinical effectiveness measurement were:

- the Beck Depression Inventory (BDI) (Hautzinger 1991),
- the Brief Symptom Inventory-18 items (BSI-18) (Franke 2000; Derogatis and Melisaratos 1983),
- the World Health Organization Disability Assessment Schedule 2.0 (WHODAS 2.0) (Üstün et al. 2010),
- the International Classification of Functioning, Disability and Health, Activities and Participation, 3 Factors (ICF-AT-3F) (Nosper 2008),
- as well as the number of days of medical treatment for 12 months before/after the intervention.

We used appropriate statistical tests to evaluate the improvement of the patients regarding the above measurements. The scale values of the statistical tests were calculated considering missing values and transformed in standard values where available. The changes between admission (*A*) and discharge (*D0*), as well as admission (*A*) and 6 months after discharge (*D6*), as well as admission (*A*) and 12 months after discharge (*D12*) were tested on significance using paired *t*-tests under consideration of a Bonferroni-correction (the following number of tests were considered: BDI: 12 tests, BSI-18: 30 tests, ICF-AT-3F: 30 tests, WHODAS 2.0: 67 tests). This has the advantage that all valid values can be considered at each particular comparison.

For the analysis of sustainability of the treatment effects according to the occupational status of the patients, only those patients, for whom we had data available at all four time points (*A*, *D0*, *D6*, *D12*)*,* were included in the analysis of variance with repeated measures. For all pairwise comparisons, Cohen’s *d* effect sizes were calculated using the Effect Size Calculator by Durham University as explained in Sect. 2.4.

#### Beck depression inventory (BDI)

The Beck Depression Inventory, BDI-score (Hautzinger 1991) was applied to measure depression severity, using a scale of 21 items by means of characteristic symptoms. The sum score is classified as follows: 0–8 = no depression, 9–13 = minimal depression, 14–19 = mild depression, 20–28 = moderate depression, and 29–63 = severe depression. A minimal clinically important difference in depression severity is defined as a minimum difference of 8 points in the BDI-score.

For the sample of 1,394 patients (cf. Table 3), the mean BDI-score was around 22.84 (moderate depression) at admission (*A*) compared to 16.24 (mild depression) at discharge (*D0*). Thus, the BDI-score significantly improved (*p* ≤  0.001) with an effect size, Cohen’s *d*, of 0.61 (medium effect). Even 12 months after discharge (*D12*), the mean BDI-score of 17.54 (mild depression) remained significantly lower (*p* < 0.001) compared to admission (*A*). However, the average BDI-scores slightly significantly worsened from discharge (*D0*) over 6 months after discharge (*D6*) to 12 months after discharge (*D12*) for all patients but they still remained in the category of mild depression which demonstrated the success of the ambulant psychiatric treatment.

**Table 3:** Paired 2-tailed *t*-tests for the BDI-scores to measure the improvement of the patients’ depression severity for the 6-week ambulant psychiatric rehabilitation program comparing different time points

**Time points comparison**	*N*	*M*_*t1*_	*SD*_*t1*_	*M*_*t2*_	*SD*_*t2*_	*t*	Sign. (*p*)	Cohen's *d*	CI *LL*	CI *UL*
*A* vs. *D0*	1394	22.84	10.63	16.24	11.09	29.38	< 0.001	0.61	0.11	0.26
*A* vs. *D6*	704	22.38	10.68	18.26	12.88	11.39	< 0.001	0.35	0.17	0.32
*A* vs. *D12*	551	22.01	10.60	17.54	12.44	10.50	< 0.001	0.39	0.11	0.26

*A* = admission, *D0/6/12* = 0/6/12 months after discharge, Cohen’s *d*: small: *d* = 0.20, medium: *d* = 0.50, large: *d* = 0.80 (Cohen 1988), *CI* confidence interval, *LL* lower limit, *UL* upper limit

Next, we investigated the BDI-scores for the three occupational groups in detail. For a subset of 321 patients (130 employed, 170 unemployed without receiving rehabilitation allowance, and 21 unemployed receiving rehabilitation allowance), both BDI-scores and occupational status were available for all four time points (*A*, *D0*, *D6*, *D12*). Analysis of variance with repeated measurements showed a significant interaction effect (*F*_[6, 634]_ = 3.176, *p* = 0.004).

Employed patients showed high effect sizes at discharge (*D0*) (*d* = 0.79), as well as 6 months after discharge (*D6*) (*d* = 0.63) and 12 months after discharge (*D12*) (*d* = 0.71). This sub-group had only minimal depression symptoms (mean BDI-score: 12.15) at 12 months after discharge (*D12*)*.*

Unemployed patients also maintained their rehabilitation success at a lower level: *A* vs. *D0*: *d* = 0.64, *A* vs. *D6*: *d* = 0.40, and *A* vs. *D12*: *d* = 0.41. However, this sub-group suffered from mild depression symptoms (mean BDI-score: 17.65) at 12 months after discharge (*D12*)*.*

For unemployed patients, who received rehabilitation allowance, we obtained a small positive effect of *d* = 0.28 at discharge (*D0*) compared to admission (*A*)*,* whereas their mean BDI-score values ranged in the category of moderate depression but improved from 26.43 to 23.04, respectively. However, this small positive effect was not confirmed at 6 months after discharge (*D6*) and 12 months after discharge (*D12*) for this sub-group.

To summarize, health care policy makers should make especially available such ambulant psychiatric rehabilitation programs to employed patients before their mental health worsens and finally causes unemployment. Furthermore, patients, who received rehabilitation allowance, necessarily need additional psychiatric treatment programs in the future.

#### Brief symptom inventory 18 items (BSI-18)

The Brief Symptom Inventory 18 items (BSI-18) score assesses the psychological distress and comorbidities in patients with different mental and somatic diseases (Franke 2000; Derogatis and Melisaratos 1983). This score contains three six-items scales (somatization, anxiety, and depression). The period of evaluation includes the well-being during the last seven days and assessment is based on a five-step likert scale from 0 = “not at all” to 4 = “extremely.” In order to interpret the results, standard values in the form of *T*-values were derived for the mean collected likert-scale values for each of the three sub-scales of the BSI-18 and for the Global Severity Index (GSI) according to the transformation guidelines of Franke (2000). The corresponding *T*-values range from 0 to 80 for assessing the severity of mental and somatic diseases as follows: *T* = 60 to *T* = 64 slightly increased, *T* = 65 to *T* = 69 considerably increased, *T* = 70 to *T* = 74 strongly increased, and *T* = 75 to *T* = 80 extremely increased. To evaluate (clinical) significant changes between two measuring time points, critical differences at single case level were available (Franke et al. 2017). Since with this statistical method less missing values are tolerated, a smaller sample size *N* was available compared to the other statistical tests, especially in the multivariate analysis. Therefore, in the analysis of sustainability regarding the treatment success, unemployed patients, who received rehabilitation allowance, were excluded.

In all three scales (somatization, anxiety, and depression) of the BSI-18-score a significant reduction of symptomatology for all ambulant rehabilitation patients (*p* ≤ 0.001) of small to medium effects was found from admission (*A*) to all three discharge time points (*D0*, *D6*, *D12*) as illustrated in Table 4. At the time of admission (*A*), most patients had considerably increased *T*-values above around 65 for all three categories (somatization, anxiety, and depression). Both between admission (*A*) and discharge after 6 months (*D6*)*,* as well as between admission (*A*) and discharge after 12 months (*D12*), a small health improvement effect was found for all patients, whereas the mean *T*-values generally remained sustainable within the range of light symptomatology (*T* = 60–64).

**Table 4:** Paired 2-tailed T-tests for the BSI-18 T-scores to measure the improvement of the patients’ symptom severity for the 6-week ambulant psychiatric rehabilitation program comparing different time points (admission, discharge)

**Time points comparison**	Sub-Scale (T-values)	*N*	*M*_*t1*_	*SD*_*t1*_	*M*_*t2*_	*SD*_*t2*_	*t*	Sign. *(p)*	Cohen's *d*	CI *LL*	CI *UL*
*A* vs. *D0*	Somatization	1450	65.96	9.03	64.08	9.43	9.96	< 0.001	0.20	0.13	0.28
	Depression	1455	66.32	8.69	62.45	9.45	19.10	< 0.001	0.43	0.35	0.50
	Anxiety	1454	67.78	9.06	65.20	9.47	12.83	< 0.001	0.28	0.21	0.35
*A* vs. *D6*	Somatization	646	65.39	8.52	64.16	9.74	3.92	< 0.001	0.13	0.03	0.24
	Depression	645	65.44	9.18	62.82	10.73	7.74	< 0.001	0.26	0.15	0.37
	Anxiety	647	67.47	8.98	64.66	10.63	8.80	< 0.001	0.29	0.18	0.40
*A* vs. *D12*	Somatization	327	65.47	8.57	63.91	9.82	3.28	< 0.001	0.17	0.02	0.32
	Depression	327	64.83	9.19	60.61	10.86	7.83	< 0.001	0.42	0.26	0.57
	Anxiety	325	66.71	9.00	62.79	11.16	7.17	< 0.001	0.39	0.23	0.54

*A* = admission, *D0/6/12* = 0/6/12 months after discharge, Cohen’s *d*: small: *d* = 0.20, medium: *d* = 0.50, large: *d* = 0.80 (Cohen 1988), *CI* = confidence interval, *LL* = lower limit, *UL* = upper limit

For a subset of 201 patients, both BSI-18 scores and the occupational status (93 employed and 108 unemployed, who did not receive rehabilitation allowance) were available for all time points (*A*, *D0*, *D6*, *D12*). The multivariate analyses of variance with repeated measures showed no significant interaction effect (*F*_[9,191]_ = 1.591, *p* = 0.120). Both employed and unemployed patients (who did not receive rehabilitation allowance), retained the obtained positive effects of the psychiatric treatment and partly increased them. For the scale somatization, a smaller effect was found in both of those employment groups. Within the group of employed patients, the scale depression showed a considerably higher treatment effect at the time point of 12 months after discharge (*D12*) compared to the discharge time points (*D0*) and (*D6*). The same trend held true for the scale anxiety, whereas employed patients gained a considerably higher treatment effect at the time point 12 months after discharge (*D12*) as well. Unemployed rehabilitation patients, who did not receive rehabilitation allowance, also obtained positive treatment effects in both scales until 12 months after discharge (*D12*) (Table 5).

**Table 5:** Effect sizes (Cohen’s *d*) for pairwise comparisons of the BSI-18-scores according to occupational status

**BSI-18 Scale**			
**Somatization**	***A*** **vs.** ***D0***	***A*** **vs.** ***D6***	***A*** **vs.** ***D12***
Employed	*ns*	0.28	0.31
Unemployed (without receiving rehabilitation allowance)	0.35	*ns*	*ns*
**Depression**	***A* vs. *D0***	***A* vs. *D6***	***A* vs. *D12***
Employed	0.36	0.43	0.64
Unemployed (without receiving rehabilitation allowance)	0.40	0.33	0.38
**Anxiety**	***A* vs. *D0***	***A* vs. *D6***	***A* vs. *D12***
Employed	0.35	0.58	0.69
Unemployed (without receiving rehabilitation allowance)	0.31	0.31	0.35

*A* = admission, *D0/6/12* = 0/6/12 months after discharge, Cohen’s *d*: small: *d* = 0.20, medium: *d* = 0.50, large: *d* = 0.80 (Cohen 1988), *ns* not significant

To summarize, the psychiatric rehabilitation patients benefited from the intervention program and could generally improve their BSI-18-scores after discharge compared to admission (*A*) and this positive effect even lasted 12 months after discharge (*D12*) for all three scales (somatization, anxiety, and depression), whereas employed patients could obtain better results. Again, the earlier in the disease progression such ambulant psychiatric rehabiliation programs are available to patients with psychiatric problems the better.

#### The World Health Organization disability assessment schedule 2.0 (WHODAS 2.0)

The World Health Organization Disability Assessment Schedule 2.0 (WHODAS 2.0) score provides a generic standardized method for measuring health and disabilities (Üstün et al. 2010). The patient’s level of functioning is captured in six domains: (1) *cognition*, (2)* mobility*, (3) *self care*, (4) *getting along*, (5) *life activities—houshold,* and (6) *participation*. The items of the domains are rated on a 5-point Likert Scale: 0 = no difficulties, 1 = mild difficulties, 2 = moderate difficulties, 3 = severe difficulties, and 4 = extreme difficulties/cannot do. Classical standard values or cut-off values are not available, while lower values indicate lower impairment of psychiatric patients.

In all six domains of the WHODAS 2.0-score, significant improvements of small positive treatment effects were found for psychiatric patients from admission (*A*) to discharge (*D0*) (cf. Table 6). As for the domains D2: Mobility (mean value of 1.11) and D3: Self-care (mean value of 0.60), ambulant psychiatric patients hardly showed any impairment at admission (*A*), only small positive treatment effects were found for those two domains.

**Table 6:** Paired 2-tailed T-tests for the WHODAS 2.0-scores to measure the improvement of the patients’ disability and health for the 6-week ambulant psychiatric rehabilitation program comparing different time points (admission and discharge)

**WHODAS 2.0:** ***A*** **vs.** ***D0***	*N*	*M*_*t1*_	*SD*_*t1*_	*M*_*t2*_	*SD*_*t2*_	*t*	Sign. *(p)*	Cohen's *d*	CI *LL*	CI *UL*
D1: Cognition—understanding and communicating	1,429	1.52	0.88	1.33	0.88	10.43	< 0.001	0.22	0.14	0.29
D2: Mobility—moving and getting around	1,427	1.11	0.92	0.98	0.91	7.62	< 0.001	0.14	0.07	0.21
D3: Self-care—attending to one's hygiene, dressing, eating, staying alone	1,426	0.60	0.71	0.51	0.66	6.17	< 0.001	0.14	0.06	0.21
D4: Getting along—interacting with other people	1,441	1.58	0.98	1.35	0.96	12.13	< 0.001	0.24	0.17	0.31
D5: Life activities—domestic responsibilities, leisure	1,433	1.75	1.12	1.62	1.12	5.72	< 0.001	0.11	0.04	0.19
D5: Life activities—work and school	452	1.87	1.28	1.69	1.23	3.41	< 0.001	0.15	0.02	0.28
D6: Participation—joining in community activities, participating in society	1,425	1.82	0.82	1.61	0.87	11.77	< 0.001	0.24	0.17	0.32

*A* = admission, *D0/6/12* = 0/6/12 months after discharge, Cohen’s *d*: small: *d* = 0.20, medium: *d* = 0.50, large: *d* = 0.80 (Cohen, 1988), *CI* confidence interval, *LL* lower limit, *UL* upper limit

Furthermore, six months after discharge (*D6*) compared to admission (*A*), stable positive improvement effects for ambulant psychiatric patients of a small extent were similarily found for the WHODAS 2.0 domains from D1 to D6 compared to the time point of discharge (*D0*). For the domain D5: Work and School, higher improvement effects of *d* = 0.41 were found for all working patients.

When comparing the WHODAS 2.0 domains scores 12 months after discharge (*D12*) to those at admission (*A*) for ambulant psychiatric patients, all six domains showed significant, in part even better improvement effects compared to discharge (*D0*) and 6 months after discharge (*D6*). For the WHODAS 2.0 domain D1: Cognition, a small effect of *d* = 0.33 was found, while for the domain D5: Domestic Responsibilities, a small effect of *d* = 0.25, and for the domain 5: Work and School, even a medium effect of *d* = 0.50 was found, respectively. For the WHODAS 2.0 domain D6: Participation, a small effect of *d* = 0.33 was obtained.

For a subset of 315 patients, both WHODAS 2.0 scores and employment status (128 employed; 166 unempolyed without receiving rehabilitation allowance, and 21 unemployed patients receiving rehabilitation allowance) were available for all time points. Multivariate analysis of variance with repeated measurements showed a significant interaction effect (*F*_[36, 592]_ = 1.626, *p* = 0.013). As for the univariate test statistics, for the domains 4: Getting along and 5: Life Activities Leisure, no significant interaction effect time*employment status was found. For the other four domains, the interaction effects were significant. The domain 5: Life Activities Work and School was calculated separately, since this domain cannot be applied to unemployed patients receiving rehabilitation allowance. However, for all discharge time points (*D0*, *D6*, *D12*)*,* employed patiented obtained the best mean scores for all WHODAS 2.0 domains, while unemployed patients, who received rehabilitation allowance, had the worst mean scores for all WHODAS 2.0 domains.

Unemployed rehabilitation patients, who did not receive rehabilitation allowance, showed more critical mean WHODAS-2.0 values than employed patients in nearly all domains, however, unemployed patients receiving rehabilitation allowance obtained by far even more critical mean WHODAS-2.0 values. Employed rehabilitation patients (when comparing to admission (*A*)) showed even better effects 6 months after discharge (*D6*) and 12 months after discharge (*D12*), as compared to discharge (*D0*) as illustrated in Table 7. Unemployed patients could maintain the positive small improvement effects in some domains (D1, D5, und D6) until 12 months after discharge (*D12*). Please note that unemployed patients receiving rehabilitation allowance represented a small group without any significant effects. Furthermore, unemployed patients, who received no rehabilitation allowance, ranged with their mean WHODAS 2.0-scores in the middle of the two groups at all discharge time points (*D0*, *D6*, *D12*). However, in most cases the mean WHODAS 2.0-values worsened from discharge (*D0*) over 6 months after discharge (*D6*) to 12 months after discharge (*D12*) but were all better compared to admission (A).

**Table 7:** Effect sizes (Cohen’s *d*) for the pairwise comparisons of the six WHODAS 2.0 score domains according to occupational status

**WHODAS 2.0 Scale**			
**D1: Cognition—understanding and communicating**	***A* vs. *D0***	***A* vs. *D6***	***A* vs. *D12***
Employed	0.32	0.41	0.51
Unemployed (without receiving rehabilitation allowance)	0.32	0.30	0.28
Unemployed (receiving rehabilitation allowance)	*ns*	*ns*	*ns*
**D2: Mobility—moving and getting around**	***A* vs. *D0***	***A* vs. *D6***	***A* vs. *D12***
Employed	*ns*	0.23	0.32
Unemployed (without receiving rehabilitation allowance)	*ns*	*ns*	*ns*
Unemployed (receiving rehabilitation allowance)	*ns*	*ns*	*ns*
**D3: Self-care—attending to one's hygiene, dressing, eating, staying alone**	***A* vs. *D0***	***A* vs. *D6***	***A* vs. *D12***
Employed	0.24	0.23	0.46
Unemployed (without receiving rehabilitation allowance)	*ns*	*ns*	*ns*
Unemployed (receiving rehabilitation allowance)	*ns*	*ns*	*ns*
**D4: Getting along—interacting with other people**	***A* vs. *D0***	***A* vs. *D6***	***A* vs. *D12***
Employed	0.32	0.31	0.32
Unemployed (without receiving rehabilitation allowance)	*ns*	*ns*	*ns*
Unemployed (receiving rehabilitation allowance)	*ns*	*ns*	*ns*
**D5: Life activities—domestic responsibilities, leisure**	***A* vs. *D0***	***A* vs. *D6***	***A* vs. *D12***
Employed	0.21	0.35	0.47
Unemployed (without receiving rehabilitation allowance)	*ns*	0.23	0.25
Unemployed (receiving rehabilitation allowance)	*ns*	*ns*	*ns*
**D5: Life activities—work and school**	***A* vs. *D0***	***A* vs. *D6***	***A* vs. *D12***
Employed	*ns*	0.48	0.58
Unemployed (without receiving rehabilitation allowance)	*ns*	*ns*	*ns*
Unemployed (receiving rehabilitation allowance)	*na*	*na*	*na*
**D6: Joining in community activities, participating in society**	***A* vs. *D0***	***A* vs. *D6***	***A* vs. *D12***
Employed	0.40	0.46	0.64
Unemployed (without receiving rehabilitation allowance)	0.27	0.27	0.34
Unemployed (receiving rehabilitation allowance)	*ns*	*ns*	*ns*

*A* = admission, *D0/6/12* = 0/6/12 months after discharge, Cohen’s *d*: small: *d* = 0.20, medium: *d* = 0.50, large: *d* = 0.80 (Cohen 1988), *na* not available, *ns* not significant

To summarize, the 6 week psychiatric rehabilitation progam could significantly improve the patient’s level of functioning measured by the WHODAS 2.0 score domains. Employed patients showed the highest functioning, while unemployed patients receiving rehabilitation allowance reached the lowest functioning. Therefore, the earlier patients were offered ambulant psychiatric rehabilitation program once they were still employed, the higher would be the success rate and the lower would be the impairment levels of the patients. Unfortunately, unemployed patients receiving rehabilitation allowance could just slightly improve their impairment levels when compared at admission (*A*) to 12 months after discharge (*D12*) for five of the six WHODAS 2.0 score domains.

#### The International Classification of Functioning, Disability and Health, Activities, and Participation, 3 Factors (ICF-AT-3F)

The International Classification of Functioning, Disability and Health, Activities, and Participation, 3 Factors (ICF-AT-3F) score conceptualizes functioning as a dynamic interaction among a person’s health condition, environmental factors, and personal factors (Nosper 2008). The three domains of the ICF-AT-3F-score include 11 items for each of the following scales: (1) *cognitive ability,* (2) *self efficacy*, and (3) s*ocial competence*. The ICF-AT-3F-score is change-sensitive and is especially suitable for clinical effectiveness measurements in the fields of psychosomatics and psychotherapy. The items have to be assessed on a five-stage Likert-scale von 0 (no or minor problem) to 5 (distinctive problem). The mean values of the three scales have to be interpreted as follows: < 0.5 = no relevant impairment, 0.5–1.0 = minor impairment, 1.0–1.5 = moderate impairment, 1.5–2.5 = considerable impairment, and > 2.5 = total impairment of activities (Nosper 2008).

In all three domains of the ICF-AT-3F score, a significant reduction of impairment of a rather small effect was found from admission (*A*) to discharge (*D0*) for all psychiatric rehabilitation patients (cf. Table 8). Six months after discharge (*D6*), a stable small effect was obtained which was minimally stronger for the scales Cognitive ability (*d* = 0.21) and Self-efficacy (*d* = 0.26). Twelve months after discharge (*D12), *this effect was even stronger for the Scales Cognitive ability (*d* = 0.28) and Self-efficacy (*d* = 0.32), respectively. In general, the mean values for all domains of the ICF 3F AT-score improved from admission (*A*) over discharge (*D0*)*,* 6 months after discharge (*D6*), to 12 months after discharge (*D12*).

**Table 8:** Paired 2-tailed T-tests for the ICF 3F AT-scores to measure the improvement of activities and participation for the 6-week ambulant psychiatric rehabilitation program comparing different time points (admission, discharge)

**ICF 3F AT** ***A*** **vs**. ***D0***	*N*	*M*_*t1*_	*SD*_*t1*_	*M*_*t2*_	*SD*_*t2*_	*t*	Sign. *(p)*	Cohen's *d*	CI *LL*	CI *UL*
Cognitive Ability	1,438	1.56	1.00	1.37	1.00	10.06	< 0.001	0.19	0.11	0.26
Self-efficacy	1,354	1.72	0.91	1.49	0.94	11.53	< 0.001	0.25	0.17	0.32
Social Competence	1,349	1.40	0.92	1.23	0.91	9.65	< 0.001	0.19	0.11	0.26

*A* = admission, *D0/6/12* = 0/6/12 months after discharge, Cohen’s *d*: small: *d* = 0.20, medium: *d* = 0.50, large: *d* = 0.80 (Cohen 1988), *CI* confidence interval, *LL* lower limit, *UL* upper limit

For a subset of 282 patients, both ICF-AT-3F scores and occupational status (116 employed; 147 unemployed without receiving rehabilitation allowance; and 19 unemployed receiving rehabilitation allowance) were available for all time points from admission (*A*) up to 12 months after discharge (*D12*). Multivariate analysis of variance with repeated measures found a significant interaction effect (*F*_[18, 2511]_ = 1.702, *p* = 0.032).

Univariate statistics obtained significant interactions for all ICF-AT-3F scales as illustrated in Table 9. Employed patients could not only maintain their positive treatment effects until 6 months (*D6*) and 12 months after discharge (*D12*) but also improved their effects. Unemployed patients, who did not receive rehabilitation allowance, could maintain their small improvement effect for the evaluation period. Patients receiving rehabilitation allowance showed no significant effect in any pairwise comparison, however, this group constituted only a small subgroup compared to the other two occupational groups. Moreover, this special group showed more critical ICF-AT-3F mean score values at admission (*A*) than employed and unemployed patients.

**Table 9:** Effect sizes (Cohen’s *d*) for the pairwise comparisons of the ICF 3F AT-score scales according to occupational status

**ICF 3F AT-Score Scale**			
**Cognitive ability**	***A* vs. *D0***	***A* vs. *D6***	***A* vs. *D12***
Employed	0.25	0.36	0.46
Unemployed (without receiving rehabilitation allowance)	0.26	0.27	0.24
Unemployed (receiving rehabilitation allowance)	*ns*	*ns*	*ns*
**Self-efficacy**	***A* vs. *D0***	***A* vs. *D6***	***A* vs. *D12***
Employed	0.37	0.37	0.54
Unemployed (without receiving rehabilitation allowance)	0.28	0.26	0.26
Unemployed (receiving rehabilitation allowance)	*ns*	*ns*	*ns*
**Social competence**	***A* vs. *D0***	***A* vs. *D6***	***A* vs. *D12***
Employed	0.24	0.26	0.29
Unemployed (without receiving rehabilitation allowance)	*ns*	*ns*	*ns*
Unemployed (receiving rehabilitation allowance)	*ns*	*ns*	*ns*

*A* = admission, *D0/6/12* = 0/6/12 months after discharge, Cohen’s *d*: small: *d* = 0.20, medium: *d* = 0.50, large: *d* = 0.80 (Cohen 1988), *ns* not significant

Similar to the findings regarding the BSI score and WHODAS 2.0 score, employed patients reached the highest functioning due to the intervention program, while patients, who were unemployed showed lower functioning measured by the ICF-AT-3F domains. Thus, these ICF-AT-3F score results reconfirmed that ambulant psychiatric rehabilitation should be early initiated once patients are still employed or at least unemployed but still capable for working.

#### The number of days of medical treatment

As a final effectiveness measure for the intervention program, we analyzed the improvement in medical treatment effort (including medication and general treatment) by comparing the number of days of medical treatment in the period of 12 months before admission, and 12 months after discharge reported by the patients. At time of admission, a total of 1,427 patients indicated the number of days of medical treatment for the period of 12 months before admission, and for 1,359 (mean = 22.40, SD = 24.76) of those, the occupational status was available too. In contrast, only data of 371 patients (mean = 19.85, SD = 23.42) were available for the period 12-months after discharge.

By performing a Wilcoxon signed-rank test (the data deviated too much from the normal distribution, thus a Wilcoxon signed-rank test was applied), we found a significant reduction in treatment days for the period of 12 months before compared to the period of 12 months after discharge, with a *p*-value of 0.001 as illustrated in Table 10. Sub-analyses according to the occupational status showed that the reduction in the number of days of medical treatment was significant both in employed (*p* = 0.02) and unemployed patients without receiving rehabilitation allowance (*p* = 0.003). However, for unemployed patients receiving rehabilitation allowance, an increase in the number of days of medical treatments was even found in the period of 12 months after discharge mean: 34 days compared to the period of 12 months before discharge (mean: 26.82 days) for the psychiatric rehabilitation program.

**Table 10:** A Wilcoxon signed-rank test to investigate differences of the number of days of medical treatment for patients depending on their employment status regarding the period of 12 months before admission compared to the period of 12 months after admission to the 6-week ambulant psychiatric rehabilitation program (adapted based on Schosser 2017)

Number of Days of Medical Treatment	*M*	*SD*	*N*	Wilcoxon signed rank test
**Number of days of medical treatment: entire sample**				
Period of 12 months before admission	22.40	24.76	1,359	*p* = 0.001
Period of 12 months after discharge	19.85	23.42	371	
**Number of days of medical treatment: employed patients**				
Period of 12 months before admission	24.47	23.07	381	*p* = 0.02
Period of 12 months after discharge	20.02	21.06	138	
**Number of days of medical treatment: unemployed patients without receiving rehabilitation allowance**				
Period of 12 months before admission	20.93	24.51	861	*p* = 0.003
Period of 12 months after discharge	17.97	23.56	207	
**Number of days of medical treatment: unemployed patients receiving rehabilitation allowance**				
Period of 12 months before admission	26.82	30.48	117	*p* = 0.455
Period of 12 months after discharge	34.00	29.57	26	

To summarize, the findings regarding the positive effects of the ambulant psychiatric rehabilitation program on the effectiveness measure of the lower patients’ medical treatment needs for the period after discharge compared to the period before admission for employed patients as well as unemployed patients without receiving rehabilitation allowance were in line with the improvements found for these two groups at/after discharge measured by the health-related psychiatric scores in Sects. 3.2.1 to 3.2.4. Again, this analysis especially indicates that unemployed patients, who were receiving rehabilitation allowance, should have undergone ambulant psychiatric treatment earlier.

### Evaluating the cost-related measures

As told in Sect. 2.2.3, a positive effect of the ambulant psychiatric rehabilitation reflected in economic terms of the 6-week ambulant psychiatric rehabilitation program is also reflected by a reduction of total costs for the period of 12 months after discharge **(*C***_***total,dis***_ **=** ***C***_***direct,dis***_ **+** ***C***_***indirect,dis***_**)** compared to the period of 12 months before the intervention **(*C***_***total,adm***_ **=** ***C***_***direct,adm***_ **+** ***C***_***indirect,adm***_**)**. If this reduction in total costs (***C***_***total,adm***_ – ***C***_***total,dis***_) is equal or higher compared to the total costs for the 6-week ambulant psychiatric rehabilitation for a patient **(*C***_***rehab***_**)**, then even direct treatment costs for the 6-week ambulant psychiatric rehabilitation of a patient could be covered within 12 months after discharge.

Firstly, we compared the direct treatment costs 12 months before admission (***C***_***direct,adm***_) to 12 months after discharge (***C***_***direct,dis***_) for our patients, then we performed a comparison of indirect costs ***C***_***indirect,adm***_ versus ***C***_***indirect,dis.***_ Finally, we focused on a total cost comparison of direct treatment and indirect costs for these 12 month periods (***C***_***total,adm/dis***_).

The positive economic effect of the ambulant psychiatric rehabilition program was reflected in a significant decrease of mean direct treatment costs (*p* ≤ 0.0001) from about 7077 Euro for the period of 12 months before admission (***C***_***direct,adm***_) to about 5790 Euro for the period of 12 months after discharge (***C***_***direct,dis***_**)** as illustrated in Table 11 using a Wilcoxon signed rank test. This effect could also be proven for both employed patients and unemployed patients without rehabilitation allowance, however not for unemployed patients with rehabilitation allowance, the latter representing the group with the most chronified course of disease and thus in need of permanent treatment. Again, the earlier the program was focused on patients, who were still employed, the lower the direct treatment costs for the period 12 months after discharge would amount to about 3996 Euro. For unemployed patient receiving rehabilitation allowance these direct costs would increase up to about 7696 Euro, whereas the sample size for this sub-group was rather low (25 patients).

**Table 11:** Direct treatment cost comparison (*C*_*direct*_) of the period 12 months before (*adm*) and 12 months after discharge for patients (*dis*) of the ambulant psychiatric rehabilitation program by a Wilcoxon signed rank test (adapted based on Schosser 2017) that compares the repeated measurements including only the samples with data available both at *adm* and *dis*

**Direct Treatment Cost Comparison**	*M*	*SD*	*N*	Wilcoxon signed rank test
**Direct Treatment Cost Comparison** ***(C***_*direct*_*)* **(Entire Sample)**				
Period of 12 months before admission ***(adm)***	7,077.03	16,305.19	1,359	*p* ≤ 0.0001
Period of 12 months after discharge ***(dis)***	5,789.50	15,372.49	301	
**Direct Treatment Cost Comparison** ***(C***_***direct***_***)* (Employed Patients)**				
Period of 12 months before admission ***(adm)***	7,149.01	13,876.29	381	*p* = 0.002
Period of 12 months after discharge ***(dis)***	3,996.16	6,997.20	111	
**Direct Treatment Cost Comparison** ***(C***_***direct***_***)* (Unemployed Patients Without Rehabilitation Allowance)**				
Period of 12 months before admission ***(adm)***	6,767.98	16,771.41	*861*	*p* = 0.001
Period of 12 months after discharge ***(dis)***	6,707.12	19,135.59	165	
**Direct Treatment Cost Comparison *(C***_***direct***_***)* (Unemployed Patients With Rehabilitation Allowance)**				
Period of 12 months before admission ***(adm)***	9,116.97	19,782.21	117	*p* = 0.626
Period of 12 months after discharge ***(dis)***	7,695.62	14,244.25	25	

Similar to the significant positive economic effects disclosed by the mean direct cost comparison of patients for the period 12 months before admission to 12 months after discharge of the psychiatric rehabilitation program, we also could confirm significant cost reductions in mean indirect costs (***C***_***indirect***_) as displayed in Table 12 by a Wilcoxon signed rank test. Again, unemployed patiens receiving rehabilitation allowance had the highest average costs of about 20,264 Euro for the period of 12 months after discharge, whereas the sample was again low with only 15 patients. It is important to mention that the indirect costs were up to 3 times higher compared to the direct costs for the patients as illustrated before in Table 11.

**Table 12:** Indirect cost comparison (*C*_*indirect*_) of the period 12 months before (*adm*) and 12 months after discharge for patients (*dis*) of the ambulant psychiatric rehabilitation program by a Wilcoxon signed rank test (adapted based on Schosser 2017**)** that compares the repeated measurements including only the samples with data available both at *adm* and *dis*

**Indirect Cost Comparison**	*M*	*SD*	*N*	Wilcoxon signed rank test
**Indirect Cost Comparison *(C***_*indirect*_*) ***(Entire Sample)**				
Period of 12 months before admission *(adm)*	20,300.77	14,824.70	1,359	*p* ≤ 0.0001
Period of 12 months after discharge *(dis)*	13,425.80	13,689.21	249	
**Indirect Cost Comparison *(C***_*indirect*_*) *** (Employed Patients)**				
Period of 12 months before admission *(adm)*	13,547.21	16,294.39	381	*p* = 0.038
Period of 12 months after discharge *(dis)*	7,441.19	10,204.45	93	
**Inirect Cost Comparison *(C***_*indirect*_*)*** (Unemployed Patients Without Rehabilitation Allowance)**				
Period of 12 months before admission *(adm)*	22,245.19	13,213.58	861	*p* ≤ 0.0001
Period of 12 months after discharge *(dis)*	16,645.60	14,407.46	141	
**Inirect Cost Comparison *(C***_*indirect*_*)*** (Unemployed Patients With Rehabilitation Allowance)**				
Period of 12 months before admission *(adm)*	27,984.12	13,085.44	117	*p* = 0.001
Period of 12 months after discharge *(dis)*	20,264.32	12,926.23	15	

Finally, we also calculated significant positive economic effects disclosed by the mean total cost comparison of patients for the period 12 months before admission to (***C***_***total,adm***_) to 12 months after discharge (***C***_***total,dis***_) of the psychiatric rehabilitation program by a Wilcoxon signed rank test (cf. Table 13). Again, highest mean total costs were induced by unemployed patients receiving rehabilitation allowance which amounted to about 37,101 Euro for the period 12 months before admission to (***C***_***total,adm***_) compared to about 28,093 Euro for the period 12 months after discharge (***C***_***total,dis***_)*,* respectively.

**Table 13:** Total cost comparison (*C*_*total*_) of the period 12 months before (*adm*) and 12 months after discharge for patients (*dis*) of the ambulant psychiatric rehabilitation program by a Wilcoxon signed rank test (adapted based on Schosser 2017) that compares the repeated measurements including only the samples with data available both at *adm* and *dis*

**Total Cost Comparison**	Mean	SD	N	Wilcoxon signed rank test
**Total Cost Comparison** ***(C****(C*_***total***_*)* **(Entire Sample)**				
Period of 12 months before admission *(adm)*	27,377.80	23,208.11	1,359	*p* ≤ 0.0001
Period of 12 months after discharge *(dis)*	19,097.17	22,756.74	249	
**Total Cost Comparison** ***(C***_***total***_***)* (Employed Patients)**				
Period of 12 months before admission *(adm)*	20,696.23	23,715.34	381	*p* = 0.005
Period of 12 months after discharge *(dis)*	11,331.30	13,556.16	93	
**Total Cost Comparison** ***(C***_***total***_***)* (Unemployed Patients Without Rehabilitation Allowance)**				
Period of 12 months before admission *(adm)*	29,013.17	22,042.85	861	*p* ≤ 0.0001
Period of 12 months after discharge *(dis)*	23,262.30	26,012.70	141	
**Total Cost Comparison** ***(C***_***total***_***)* (Unemployed Patients Rehabilitation Allowance)**				
Period of 12 months before admission *(adm)*	37,101,10	24,474.90	117	*p* = 0.031
Period of 12 months after discharge *(dis)*	28,093,46	23,181.65	15	

To evaluate the overall societal cost benefit of the intervention program, we assessed that the reduction in total costs (***C***_***total,adm***_ – ***C***_***total,dis***_) amounting to on average 8280.63 Euro was higher compared to the total costs for the 6-week ambulant psychiatric rehabilitation for a patient **(*C***_***rehab***_**)** ranging between 3731.74 Euro and 4390.23 Euro. Thus, we found that direct treatment costs for the 6-week ambulant psychiatric rehabilitation of a patient **(*C***_***rehab***_**)** could be easily covered within 12 months after discharge once a total societal cost perspective was considered (***C***_***total,adm***_ – ***C***_***total,dis***_). Even additional cost savings of up to over 5000 Euro could be achieved which were highest for employed patients, followed by unemployed patients receiving rehabilitation allowance due to their high direct treatment costs and high indirect costs for productivity loss. If only direct costs for the period of 12 months before admission/after discharged were taken into account, then costs per patient for the 6-week ambulant psychiatric rehabilitation program (***C***_***rehab***_) could only be partly covered with an average of about 1287.53 Euro within 12 months after discharge. However, according to the recommendations of the panel on cost-effectiveness in health and medicine both direct and indirect costs of an intervention program should be considered (Weinstein et al. 1996).

## Conclusions and policy implications

Effectiveness of the 6-week ambulant psychiatric rehabilitation program was assessed both in terms of clinical effectiveness indices for measuring depression severity, symptom burden, and functioning to document the patients’ health improvement, in terms of economic effectiveness such as the number of sick leave days, as well as in terms of cost measures.

For most of the clinical effectiveness indices, we found significant improvement of the patients after discharge (*D0*) and even onwards (*D6*, *D12*) compared to admission (*A*) depending on the occupational status. Generally, employed patients compared to unemployed patients better improved after discharge (*D0*), while unemployed patients receiving rehabilitation allowance mostly reached the worst scores at discharge and onwards (*D6*, *D12*). Thus, the earlier this 6-week rehabilitation programm is iniated for patients who are still employed or unemployed without receiving rehabilitation allowance, the higher will be the clinical effectiness.

For the Beck Depression Inventory, BDI score (Hautzinger 1991) to measure depression severity, this trend was significantly confirmed. Especially, the unemployed patients receiving rehabilitation allowance had the highest depression severity compared to the patients who were either employed or unemployed without receiving rehabilitation allowance.

The Brief Symptom Inventory-18 (BSI-18) score (Franke 2000; Derogatis and Melisaratos 1983), which is used to assess the psychological distress and comorbidities in patients with different mental and somatic diseases, showed a significant reduction of symptomatology of small to medium effects between admission (*A*) and discharge (*D0*). Unemployed patients, who did not receive rehabilitation allowance, obtained their positive treatment effect in two scales until 12 months after discharge (*D12*). Interestingly, within the group of employed patients, the scales depression and anxiety showed considerably higher effects 12 months after discharge (*D12*) than at discharge (*D0*) and 6 months after discharge (*D6*). Therefore, health care policy makers should especially make available such ambulant psychiatric rehabiliation programs to employed patients before their mental health worsens and finally causes unemployment.

Significant improvements were presented after discharge (*D0*) and even onwards (*D6*, *D12*) compared to to admission *(A)* for all patients’ domains of the World Health Organization Disability Assessment Schedule 2.0 (WHODAS 2.0) score (Üstün et al. 2010), which provides a standardized method for measuring health and disabilities across all diseases, whereas the success depended on occupational status of the patients.

Patients reached better functioning scores when measured by the ICF-AT-3F score (International Classification of Functioning, Disability and Health, Activities and Participation, 3 Factors) (Nosper 2008), which conceptualizes functioning as a dynamic interaction among a person’s health condition, environmental factors, and personal factors. Again, employed patients demonstrated higher functioning than unemployed patients, and especially than those who were receiving rehabilitation allowance.

We further found a significant reduction in the number of days of medical treatment (including treatment by general practitioner, psychiatrist, psychotherapist, psychologist; inpatient treatment; and psycho-pharmacological treatment) for the period of 12 months before admission compared to the period of 12 months after discharge reported by the patients. A significant reduction of productivity loss (sick leave days per patient) from the period of 12 months before admission compared to the period 12 months after discharge was proven for all occupational patient groups.

For the cost measures, both direct tangible treatment and medication costs and indirect tangible costs based on the productivity loss measured in non-working days of the patients were considered. Both direct costs and indirect costs significantly dropped from the period of 12 months before admission compared to the period of 12 months after discharge.

Higher costs savings could be found in the category of indirect costs. From a clinical point of view, patients treated in psychiatric rehabilitation programs usually have to continue treatment (in most cases for at least several months to a year) to gain further stabilization. Besides, for some patients, the ambulant rehabilitation program was the first extensive treatment of their mental health problem, since before rehabilitation they either had received no treatment, or only psycho-pharmacological treatment by a general practitioner but among others no psychotherapeutic treatment. Therefore, it was not surprising that the direct costs could not sharply drop after rehabilitation compared to before rehabilitation.

For total costs (direct and indirect costs), patients occurred mean costs of about 27,378 Euro for the period of 12 months before admission compared to mean costs of about 19,097 Euro for the period of 12 months after discharge which is a cost saving of about 8281 Euro per patient. Highest savings could be achieved for employed patients, followed by the group of unemployed patients receiving rehabilitation allowance. Thus, the direct treatment costs for the 6-week ambulant psychiatric rehabilitation program for a patient (ranging from about 3732 Euro to 4390 Euro) could be easily covered within 12 months after discharge once a total societal cost perspective was considered.

Even additional cost savings of up to over 5000 Euro could be achieved per patient which were highest for employed patients, followed by unemployed patients receiving rehabilitation allowance both due to their high direct treatment costs and high indirect costs for productivity loss. However, this finding of impressive cost savings in the latter group has to be interpreted with caution, since only for 15 of the 117 rehabilitants receiving rehabilitation allowance, information on occupational status was available at 12 months after discharge (*D12*).

To summarize, the 6-week ambulant psychiatric rehabilitation program under investigation was clearly successful in terms of clinical effectiveness measures and in terms of cost measures for both employed and unemployed patients, however with ambiguous outcomes especially in terms of clinical effectiveness for unemployed patients receiving rehabilitation allowance. Nevertheless, the cost savings of the latter group were impressing and clearly higher than in unemployed rehabilitants without rehabilitation allowance. This effect was due to that 15% of patients receiving rehabilitation allowance that returned to work after rehabilitation. Thus, it is essential for health care policy makers to provide this 6-week ambulant rehabilitation program to employed patients as early as possible when they are showing first symptoms before they will lose their work and will even become incapable for working. However, one should also consider the equity of interventions programs besides the ordinary cost-effectiveness criteria to avoid too much discrimination among different patient groups (Aday et al. 1999). Equity criteria can include procedural equity (dimensions: deliberative justice, distributive justice, distributive and social justice, and social justice) and substantive equity (dimension: health) which could be accounted for by suitable equity indicators.

The limitations of the current study are that all calculations are based on self-report questionnaires, and, particularly, 12 months after discharge (*D12*) only data on a subset of patients were available, since a high percentage did not return the catamnesis questionnaires. This phenomenon is well known and is probably caused by the fact that rehabilitants were neither obligated to fill in catamnesis questionnaires, nor did they get any financial compensation. A further limitation may be that the study does not control for the well-known “regression to the mean effect” (cf. Morton and Torgerson 2003) because patients tend to recover from many diseases even without treatment and at the peak of the symptoms they often seek treatment. This effect could only be investigated by including a standardized control group which could be a topic for further research. Another limitation is that we could only conduct an uncontrolled repeated measures study. Furthermore, cost data per patient could also be only based on average costs and questionnaires as we were not allowed to obtain health care data per patient from the social security system due to data security limitations.

Further research would highly benefit from both more detailed data per psychiatric rehabilitation patient related to health care, occupation, and social security during as well as before/after the ambulant treatment. Such improved data could be used for establishing patient pathway models which then could be further optimized by multi-criteria approaches. In addition, data mining techniques, big data analytics, and machine learning applications open new fields for better analyzing various characteristics of psychiatric patients and their disease progression depending on the kind, timing, and cost-effectiveness of different treatment options.

## References

[CR1] Aday LA, Begley CE, Lairson DR, Slater CH, Richard AJ, Montoya ID (1999). A framework for assessing the effectiveness, efficiency, and equity of behavioral healthcare. Am J Manag Care.

[CR3] Armijo J, Méndez E, Morales R, Schilling S, Castro A, Alvarado R, Rojas G (2013). Efficacy of community treatments for schizophrenia and other psychotic disorders: a literature review. Front Psychiatry.

[CR4] Atroszko PA, Demetrovics Z, Griffiths MD (2020). Work addiction, obsessive-compulsive personality disorder, burn-out, and global burden of disease: implications from the ICD-11. Int J Environ Res Public Health.

[CR5] Barrett NM, Gill KJ, Pratt CW, Roberts MM (2013). Psychiatric rehabilitation.

[CR6] Beecham J (2014). Annual research review: child and adolescent mental health interventions: a review of progress in economic studies across different disorders. J Child Psychol Psychiatry.

[CR7] bestNET Information-Service GmbH (2016) Costs for psychotherapists. https://www.psyonline.at. Accessed 22 Aug 2019

[CR8] Brailsford S, Harper P (2008). OR in health. Eur J Oper Res.

[CR9] Brailsford S, Vissers J (2011). OR in healthcare: a European perspective. Eur J Oper Res.

[CR10] Brandeau ML, Sainfort F, Pierskalla WP (2004). Operations research and health care: a handbook of methods and applications.

[CR11] Bundesministerium für Arbeit, Soziales, Gesundheit und Konsumentenschutz (2019) *Rehabilitationsgeld und medizinische Rehabilitation, Bericht über den Zeitraum der Jahre 2014 bis 2018 und Schwerpunkt auf das Jahr 2018*. Bundesministerium für Arbeit, Soziales, Gesundheit und Konsumentenschutz, Vienna, Austria

[CR12] Camacho EM, Shields GE (2018). Cost-effectiveness of interventions for perinatal anxiety and/or depression: a systematic review. BMJ open.

[CR13] Cohen J (1988). Statistical power analysis for the behavioral sciences.

[CR14] Cohen J (1992). A power primer. Psychol Bull.

[CR15] Cyranowski JM, Frank E, Young E, Shear MK (2000). Adolescent onset of the gender difference in lifetime rates of major depression: a theoretical model. Arch Gen Psychiatry.

[CR16] Derogatis LR, Melisaratos N (1983). The brief symptom inventory: an introductory report. Psychol Med.

[CR17] Franke GH, Jaeger S, Glaesmer H, Barkmann C, Petrowski K, Braehler E (2017). Psychometric analysis of the brief symptom inventory 18 (BSI-18) in a representative German sample. BMC Med Res Methodol.

[CR18] Franke GH (2000) BSI Brief Symptom Inventory von L.R. Derogatis (Kurzform der SCL-90-R) - Deutsche Version: Manual. Beltz, Göttingen, Germany

[CR19] Haberfellner EM, Jungmayr J, Grausgruber-Berner R, Grausgruber A (2008). Stationäre medizinische Rehabilitation von Patienten mit psychiatrischen oder psychosomatischen Erkrankungen in Österreich - eine katamnestische Studie. Rehabilitation.

[CR20] Hautzinger M (1991). The Beck Depression Inventory in clinical practice. Nervenarzt.

[CR21] IBM Corporation (2017) IBM SPSS Statistics for Windows, Version 25.0, IBM Corporation, Armonk, United States of America

[CR22] Knapp M, Wong G (2020). Economics and mental health: the current scenario. World Psychiatry.

[CR23] König H, König H, Konnopka A (2020). The excess costs of depression: a systematic review and meta-analysis. Epidemiol Psychiatric Sci.

[CR24] Kola L (2020). Global mental health and COVID-19. Lancet Psychiatry.

[CR25] Konnopka A, König H (2020). Economic burden of anxiety disorders: a systematic review and meta-analysis. Pharmacoeconomics.

[CR26] Landeskrankenhaus Steyr (2016) Costs of inpatient treatment. Landeskrankenhaus Steyr, Steyr

[CR27] Lenhard W, Lenhard A (2016). Calculation of effect sizes. Psychometrica.

[CR28] Mallor F, Brailsford S, Rauner M, Azcarate C (2018). Operational research applied to health services: finding better health-care decisions in new oceans of health data. Oper Res Health Care.

[CR29] Morton A, Rauner M, Zaric G (2016). Special Issue on Healthcare, introduction to the special issue. EURO J Decis Process.

[CR30] Morton V, Torgerson DJ (2003). Effect of regression to the mean on decision making in health care. BMJ.

[CR31] Na EJ, Kang NI, Kim MY, Cui Y, Choi HE, Jung AJ, Chung YC (2016). Effects of community mental health service in subjects with early psychosis: one-year prospective follow up. Community Ment Health J.

[CR32] Nosper M (2008) ICF AT-50: Entwicklung eines ICF-konformen Fragebogens für die Selbstbeurteilung von Aktivitäten und Teilhabe bei psychischen Störungen. In: Deutsche Rentenversicherung Bund, editor. 17. Rehabilitationswissenschaftliches Kolloquium; 3.-5–3.2008; Bremen; pp 127–128

[CR33] Patton GC, Sawyer SM, Santelli JS (2016). Our future: a Lancet commission on adolescent health and wellbeing. Lancet.

[CR34] Rabenstein R, Pintzinger N, Knogler V, Kirnbauer V, Lenz G, Schosser A (2015). Wirksamkeit eines ambulanten, verhaltenstherapeutisch orientierten Rehabilitationsprogramms—eine Wartelistenkontrollgruppenstudie. Verhaltenstherapie.

[CR35] Rauner MS, Vissers JM (2003). OR applied to health services: planning for the future with scarce resources. Eur J Oper Res.

[CR36] Rauner MS, Behrens DA, Wild C (2005). Quantitative decision support for health services. CEJOR.

[CR37] Reiter D, Fülöp G, Gyimesi M, Nemeth, C (2012) Rehabilitationsplan 2012. Gesundheit Österreich, Forschungs- und Planungs-GesmbH, Vienna, Austria

[CR38] Roessler W (2006). Psychiatric rehabilitation today: an overview. World Psychiatry.

[CR39] Saanich News (2018) Campaign makes connection with mental health support. https://www.saanichnews.com/news/campaign-makes-connection-with-mental-health-support. Accessed 15 Jan 2018

[CR40] Saxena S, Carlson D, Billington R, Orley J (2001). The WHO quality of life assessment instrument (WHOQOL-BREF): the importance of its items for cross-cultural research. Qual Life Res.

[CR41] Senft B, Nosper M, Leonhart R Platz T (2013) Medizinische Rehabilitation psychisch Kranker in Österreich—Auf dem Weg zu ICF-orientierter Evaluation. In: Deutsche Rentenversicherung Bund (Hrsg.), 22. Rehawissenschaftliches Kolloquium. Teilhabe 2.0 - Reha neu denken? (DRV-Schriften, Bd. 101, Bd. 101, S. 465–467)

[CR42] Senft B, Fischer-Hansal D, Schosser A (2020) Was heißt signifikant—geheilt oder nur etwas besser? Ein Vergleich verschiedener Berechnungsmethoden zur Bewertung von Veränderungen der depressiven Symptomatik bei ambulanten Rehabilitanden. Neuropsychiatrie: Klinik, Diagnostik, Therapie und Rehabilitation: Organ der Gesellschaft Österreichischer Nervenarzte und Psychiater, 11(3):103. 10.1007/s40211-020-00343-z 10.1007/s40211-020-00343-z 32162108

[CR44] Schneider F, Dreer E (2013). Volkswirtschaftliche Analyse eines rechtzeitigen Erkennens von Burnout.

[CR45] Schosser A (2017) Cost-effectiveness analysis of an ambulant psychiatric rehabilitation program in Austria. Master Thesis, Medical University of Vienna, Austria

[CR46] Schöffski O, von der Schulenburg JMVD (2008). Gesundheitsökonomische evaluationen.

[CR47] Sigmund Freud University (2016). Costs for psychologists.

[CR48] Sozialrechts-Änderungsgesetz 2012, BGBl. I Nr. 3/2013. https://www.ris.bka.gv.at/eli/bgbl/I/2013/3/20130110. Accessed 22 Aug 2019

[CR50] Statistik Austria (2017) Income Data for Austria 2014–2016. Statistik Austria, Vienna, Austria

[CR51] Statistik Austria (2019) ÖNACE 2008 Klassifikationsdatenbank. http://www.statistik.at/KDBWeb/kdb_VersionAuswahl.do. Accessed 22 Aug 2019

[CR52] Steffanowski A, Löschmann C, Schmidt J, Wittmann WW, Nübling R (2007). Meta-Analyse der Effekte stationärer psychosomatischer Rehabilitation: Mesta-Studie.

[CR53] Sullivan LM (2008). Repeated measures. Circulation.

[CR54] Sunkel C, Haring R, Kickbusch I, Ganten D, Moeti M (2021). Global burden of mental illness. Handbook of global health.

[CR55] Üstün TB, Kostanjsek N, Chatterji S, Rehm J (eds) (2010) Measuring health and disability: manual for WHO disability assessment schedule WHODAS 2.0. World Health Organization, Geneva

[CR56] Weber GW, Blazewicz J, Rauner M, Türkay M (2014). Recent advances in computational biology, bioinformatics, medicine, and healthcare by modern OR. CEJOR.

[CR57] Weinstein MC, Siegel JE, Gold MR, Kamlet MS, Russell LB (1996). Recommendations of the panel on cost-effectiveness in health and medicine. J Am Med Assoc.

[CR58] Wiener Gebietskrankenkasse (2016) Costs for psychiatrics. Wiener Gebietskrankenkasse, Vienna, Austria

[CR59] Wilcoxon F (1945). Individual comparisons by ranking methods. Biom Bull.

[CR60] World Health Organization (1993). The ICD-10 classification of mental and behavioural disorders: diagnostic criteria for research.

[CR61] World Health Organization (2013). Mental Health Action Plan 2013–2020.

[CR62] Yoon IA, Slade K, Fazel S (2017). Outcomes of psychological therapies for prisoners with mental health problems: a systematic review and meta-analysis. J Consult Clin Psychol.

[CR63] Zaric GS (2013). Operations research and health care policy.

